# Aerial Video Trackers Review

**DOI:** 10.3390/e22121358

**Published:** 2020-11-30

**Authors:** Jinlu Jia, Zhenyi Lai, Yurong Qian, Ziqiang Yao

**Affiliations:** College of Software, Xinjiang University, Urumqi 830000, China; jjj@stu.xju.edu.cn (J.J.); feng@stu.xju.edu.cn (Z.L.); yzq@stu.xju.edu.cn (Z.Y.)

**Keywords:** aerial video, visual target tracking, siamese network, depth feature

## Abstract

Target tracking technology that is based on aerial videos is widely used in many fields; however, this technology has challenges, such as image jitter, target blur, high data dimensionality, and large changes in the target scale. In this paper, the research status of aerial video tracking and the characteristics, background complexity and tracking diversity of aerial video targets are summarized. Based on the findings, the key technologies that are related to tracking are elaborated according to the target type, number of targets and applicable scene system. The tracking algorithms are classified according to the type of target, and the target tracking algorithms that are based on deep learning are classified according to the network structure. Commonly used aerial photography datasets are described, and the accuracies of commonly used target tracking methods are evaluated in an aerial photography dataset, namely, UAV123, and a long-video dataset, namely, UAV20L. Potential problems are discussed, and possible future research directions and corresponding development trends in this field are analyzed and summarized.

## 1. Introduction

Visual target tracking is an important topic in the field of computer vision. Its purpose is to accurately locate, identify and track the target after obtaining continuous images through the collector. An overview of research progress and visualization achievements at home and abroad reveals that visual target-tracking technology has unique social application value in terms of convenience, high efficiency, safety, reliability, high cost performance and low energy consumption [1] in the fields of medical diagnosis, human-computer interaction, public safety [2], video surveillance and posture estimation [3].

However, there are some differences between aerial target tracking technology and standard ground target tracking technology. The differences of among aerial photography instruments, environments and target states, which lead to high information content, multiple heterogeneity and high dimensionality of aerial photography images or videos. Available image processing algorithms such as image denoising [4], image enhancement [5] and image mosaicking [6] can satisfy the real-time processing requirements of aerial image target recognition, but difficult problems and challenges remain in the realization of target tracking, including the following.

### 1.1. Target Specificity

Aerial photography instruments have light sensitivities that differ among targets and are limited by their own flight height. In aerial photography images, there are often targets that are visible to the naked eye but have a small pixel size and image objects that blur or resemble the actual background color texture [7,8]. This study conducts a classification based on the characteristics of aerial photography targets according to the following six types:

1. Dim small targets: Targets for which the imaging size is relatively small due to the shooting angle and shooting distance-namely, targets for which the imaging size is less than 0.12% of the total number of pixels [9].
2. Weakly fuzzy targets: Targets for which the image is blurred due to the exposure time or flight jitter.
3. Weak-contrast targets: In a recognition environment with low noise and a low signal-to-noise ratio (SNR), the recognition target and moving background are similar in terms of color features and texture features. Hence, the contrast between the recognition target and the background is low, and the texture feature is not readily identified, but there is no missing target category.
4. Occluded targets: Targets that are temporarily occluded by the complex environmental background or are hidden for a long time during aerial photography tracking.
5. Fast-moving targets: Targets that exhibit dodging, fleeing and fast movement, which include image debris that is caused by the shaking of the UAV fuselage, obstacle avoidance and the influence of wind speed.
6. Common targets: Targets with normal behavior and clear images.

### 1.2. Background Complexity

Aerial photography can be roughly divided into three types: urban architectural landscape (e.g., urban road, urban building, and large-scale event site) photography, suburban open area (plain, grassland, and open area in an urban suburb) photography and complex and harsh environment scene (desert, mountain, gully and natural disaster site) photography. Due to the diverse environment, the pixel values of aerial photography targets and backgrounds are relatively low, namely, the texture features, spatial features and color features of the background differ substantially, which causes strong interference with aerial photography targets, especially in the case of complex environmental changes, sudden unknown static or mobile threats to aerial photography equipment, and other challenges in aerial photography. This paper summarizes the methods for overcoming target occlusion that is caused by a high-resolution pixel ratio in aerial photography and high feature complexity dimension.

### 1.3. Tracking Diversity

Aerial image acquisition equipment results in a variety of data forms, which include ordinary red, green, blue (RGB) color images (visible light images), infrared thermal images (gray images), GPS navigation information and acquisition equipment number information. Therefore, by combining various data features, the identification and tracking of occluded targets and weak targets can be realized. By using a single-UAV working mode or multi-UAV collaborative tracking mode, the number of available target features (spatial three-dimensional and multiangle features) can be increased to increase the tracking accuracy and tracking success rate. However, problems such as collaborative path planning, data normalization and image edge calculation are encountered.

According to the characteristics of the aerial video shooting target, this study conducts classification comparison of target-tracking methods and identification of the characteristics of various methods and usage scenarios. The main contributions of this paper can be described as follows.

- We conduct a comprehensive benchmark test of aerial video trackers based on handcrafted feature and deep learning.
- We take the target scale and definition as the classification criteria and conduct a complete comparative analysis of the three tracking schemes.
- We benchmark 20 trackers based on handcrafted feature, depth Feature, siamese network and attention mechanism.
- We compare the performance of the tracker in various challenging environments, so that relevant researchers can better understand the research progress on aerial video tracking.

The remainder of this paper is organized as follows. In Section 1, we explain the definition of aerial video target tracking from three perspectives: the target type, the shooting background and the tracking method. In Section 2, we compare the relevant datasets that can be used for aerial target tracking. In Section 3, we relevant tracking methods are introduced from three aspects: ordinary targets, weak targets and moving targets. In Section 4, we investigate and compare the structures of neural network trackers. In Section 5, we show the evaluation results of different trackers under UAV123 and UAV20L standards through experimental comparison and discuss the comparison between different trackers and the potential problems of aerial target tracking. In Section 6, we discuss the future research direction of aerial target tracking.

## 2. Aerial Video Datasets

Due to differences in the sensors of aerial photography equipment, parameters may vary among datasets [10]. Any single-frame image in a dataset contains multiple targets, but the frequency of the targets is not stable, and the target position and attitude change with the shooting angle. Therefore, although various traditional aerial photography datasets can reflect the application requirements of the real world, their application degree is typically low.

Aerial photography data are typically acquired by low-altitude drones. The number of videos in Table 1 represents the number of videos in the dataset, shortest video frames represents the number of frames in the video sequence with the fewest frames in the dataset, longest video frames represents the number of frames in the video sequence with the most frames in the dataset, total video frames represents the sum of the numbers of frames of all the video sequences in the dataset, and average video frames is obtained by dividing the total number of frames in the dataset by the number of videos. OTB and VOT are common target datasets, which are suitable for short-term tracking. The LaSOT dataset, consisting of 3.52 million manually annotated images and 1400 videos, is focused on long-term tracking and is by far the largest target dataset with dense annotation. However, these datasets contain substantial amounts of nonaerial target information and are not suitable for aerial target tracking. UAV123, ALOV300++, and Temple Color 128 are excellent special aerial photography datasets with rich types. Among them, the objects, such as dancers, completely transparent glass, octopuses, birds and camouflaged soldiers, exhibit occlusion, complete occlusion and sudden movement of the target, which are more in line with practical scenarios. UAV123 has a wide variety of scenes, which include urban landscapes, roads, buildings, sites, beaches and ports. The targets include cars, trucks, ships, people, groups and air vehicles, and the activities include walking, cycling, water skiing, driving and swimming. The long-term complete and partial occlusions of the target, scale changes, light changes, view changes, background clutter, camera motion and other effects are labeled. UAV123 has recently become increasingly popular due to its practical applications, such as navigation, wildlife surveillance, and crowd surveillance.

## 3. Traditional Target Tracking Algorithm

The combination of UAV with infrared equipment can solve the tracking problem of weak targets and hidden targets [21]. However, due to the high data feature dimensions, it is not suitable for the tracking analysis of fast-moving targets and exhibits low real-time performance. Many challenges remain in the real-time tracking of aerial photography. In addition, target loss that is caused by target deformation and different scales is an urgent problem to be solved. This section summarizes the problem in terms of the target category in the problem definition. Weak targets are defined in Section 1.

### 3.1. Common Targets

The traditional template-matching target tracking strategy is to construct a tracer based on sparse representation. The best candidate box can be identified by using template matching, but the background and target cannot be distinguished well. Reference [22] proposes the adaptive structural local sparse appearance (ASLA) algorithm, which increases the tracking accuracy and reduces the influence of occlusion by aligning the pooling operation on sparse code. Next, augmented quantum space learning and sparse representation are adopted in the update module to address drift and partial occlusion.

Various target trackers realize satisfactory short-time tracking performance, whereas others realize satisfactory long-time tracking performance. In Reference [23], the MUlti-Store Tracker (MUSTer) algorithm combines these two types of trackers—For short-time tracking, a powerful integrated correlation filter (ICF) method is used for short-term storage. The use of key-point matching tracking and random sample consensus [24] estimation in integrated long-term modules enables the integration of long-term memory and provides additional information for output control.

To overcome the high data feature dimensions, Reference [25] utilized the principal component analysis and scale-invariant feature transform (PCA-SIFT) algorithm, which improved SIFT and introduced PCA to reduce the dimensionality of aerial target features. Due to the loss of information in dimensionality reduction, this method is suitable for processing only clear aerial video images of targets. To overcome background interference and background shade, Reference [26] uses the appearance of the target and the background environment to build a tracker from two angles. The tracker is robust to changes in the appearance of the target during tracking. First, background patch information and foreground patch information are obtained, and multiangle information is associated through camera calibration. An adaptive model update strategy based on response distribution and prior tracking results is used to reduce the possibility of model drift and enhance tracking stability. Reference [27] designed a robust tracker that is based on a key patch sparse representation and designed patches for the occlusion part. First, using patch sparsity, patches are obtained from known images, and scores are provided. Second, key patches are selected according to the position and occlusion scenario, and corresponding contribution factors are designed for sampling patches to emphasize the contributions of selected key patches. This method increases the accuracy of partial occluded target tracking.

### 3.2. Weak Targets

In weak target tracking, two main challenges are encountered. First, the distance between the aerial photography equipment and the tracking target is relatively large, and the target occupies a relatively low percentage of pixels on the imaging plane and is vulnerable to interference by various types of noise clutter, thereby resulting in a missing target or target loss. Second, environmental factors (complex background, wind speed, and equipment jitter) lead to target blur and target loss. In this paper, weak targets, weakly contrasted targets and weak blurred targets are discussed and analyzed.

#### 3.2.1. Dim Small Targets

To reduce the omission rate of dim small targets and increase the tracking accuracy, the relative local contrast measure (RLCM) multiscale detection algorithm was used in Reference [28]. The algorithm calculates the multiscale RLCM for each pixel of the original infrared image to enhance the real target and suppress all types of interference (such as high brightness background, complex background edges and pixel-sized noise with high brightness). An adaptive threshold is used to extract the real target. Formulas (1)–(3) calculates the RLCM of the center pixel of the center cell at each location.

(1) $$RLCM=\min(\frac{Imean_{0}}{Imean_{i}}Imean_{0}-Imean_{0}),$$

(2) $$Imean_{0}=\frac{1}{K_{1}}\sum_{j=1}^{K_{1}}G_{0}^{j},$$

(3) $$Imean_{i}=\frac{1}{K_{2}}\sum_{j=1}^{K_{2}}G_{i}^{j},i=1,2,3,...,8.$$

where $\frac{Imean_{0}}{Imean_{i}}$ can be understood as an enhancement factor for the central cell [that is, cell(0)] in the $i_{th}$ direction, and $Imean_{0}$ and $Imean_{i}$ denote the average gray values of the $K_{1}$ or $K_{2}$ max pixels in cell(0) and cell(i), respectively. $K_{1}$ and $K_{2}$ are the numbers of maximal gray values that are considered, and $G_{0}^{j}$ and $G_{i}^{j}$ are the $j_{th}$ maximal gray values of cell(0) and cell(i), respectively.

**Figure 1:**
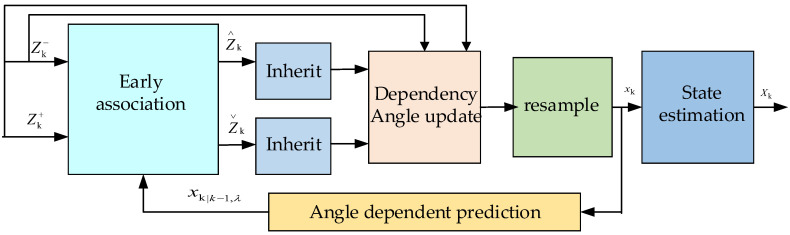
Probability hypothesis density particle filter framework calculation process. At time k, strong detection Z${}_{k}^{+}$ and weak detection Z${}_{k}^{-}$ are associated with the predicted state that is calculated from the predicted particles. After the early association, two detection subsets are used for tracking. Detection ${\overset{\wedge }{Z}}_{k}$ inherits the identity of the corresponding trajectory and is used to track the state, and ${\overset{\vee }{Z}}_{k}$ are unassociated strong detections and are used to initialize new states. After updating and resampling of the perspective, particle x${}_{k}$ is used to estimate state X${}_{k}$.

In Reference [29], an online multitarget tracker was designed by using high confirmations (strong detections) and low confirmations (weak detections) in the framework of the probability hypothesis density particle filter, which performed well in terms of tracking accuracy, number of missing targets and speed. The calculation flowchart is presented in Figure 1.

Strong detections are used to propagate target tags and promote target initialization, whereas weak detections are used only to support label propagation. Early association (EA) is executed prior to the trust angle update phase to reduce the extensive computational cost that is incurred by the labeling process. The federated data ${\overset{\wedge }{Z}}_{k}$ inherit the corresponding identity information and are only used to track the status. After the EA phase, weak target detections that are not connected are discarded, while unassociated strong detections ${\overset{\vee }{Z}}_{k}$ are retained for the initialization of new particles. Strong detection generates new particles, as expressed in Formula (4), where N(·) is a Gaussian distribution, x${}_{k}^{i}$ represents the relative weight of each new particle, and $X_{k,\lambda }^{i}$ is the $i_{th}$ particle. Strong detection generates new detection particles independently modeled from the estimated state according to the function N(·) and dynamically updated based on parameters such as the detection size and video frame rate using covariance matrix ∑. Moreover, unassociated strong detections initialize a new particle, as expressed in Formula (5), where |·| is the specified set and $Z_{k}$ represents combined detections. $\sum_{k}$ is a standard deviation matrix that changes with time. It defines the relationship between the target detection tracking box and the weight of the new particles. These values can be learned from the training set, and state evaluation is conducted, as expressed in Formula (6), where each state $x_{k,\lambda }\in X_{k}$ is estimated as the average of all resampled particles sharing the same identity.

(4) $$X_{k,\lambda }^{i}∼p_{k}\left(X_{k,\lambda }^{i}|Z_{k}^{+}\right)=\frac{1}{\left|Z_{k}^{+}\right|}\sum_{\forall z_{k}^{+}\in Z_{k}^{+}}N\left(X_{k,\lambda }^{i};Z_{k}^{+},\sum \right),$$

(5) $$X_{k,\lambda }^{i}∼p_{k}\left(X_{k,\lambda }^{i}|\overset{\vee }{z_{k}}\right)=\frac{1}{\left|\overset{\vee }{z_{k}}\right|}\sum_{\forall \overset{\vee }{z_{k}}\in \overset{\vee }{Z_{k}}}N\left(X_{k,\lambda }^{i};\overset{\vee }{z_{k}},\sum_{k}\right),$$

(6) $$X_{k,\lambda }=\frac{1}{\left|\chi_{k,\lambda }\right|}\sum_{\forall X_{k,\lambda }^{i}\in \chi_{k,\lambda }}X_{k,\lambda }^{i}.$$

Reference [30] realizes the feature binding of the target’s grayscale and spatial relation via compressed perception, thereby constructing a gaussian target to overcome high similarity between the small target and the background noise. Reference [31] combines particle swarm optimization (PSO) and a particle filter to optimize the sampling process of the particle filter to overcome small target feature poverty. In addition, the algorithm introduces the local PSO reset method to overcome the particle collapse problem in the particle filter for multitarget detection and tracking.

#### 3.2.2. Weak Blurred Targets

The infrared detection system is typically used to find and track weak blurred targets. Reference [32] applied the Wiener filter to the processing of the original infrared image. First, motion blur is processed, and noise interference is suppressed. The gradient method is then used to sharpen the processed image to enhance the target edge. This method can substantially reduce the motion blur, increase the image quality and enhance the performance of the detection system. Reference [33] constructed a nonlinear blurred core with multiple moving components. A blind deconvolution technique that used a piecewise linear model was introduced to estimate the unknown kernels. This method is combined with noise reduction technology that is based on wavelet multiframe decomposition and the peak signal-to-noise ratio (PSNR). This algorithm is highly effective in accurately identifying various blurred cores and provides important research strategies for image defuzzing. Reference [34] proposes a new motion blurred computing method for ray tracking. This method provides analysis data of the blurred visibility of each ray motion and considers the time dimension. The algorithm can use any standard ray tracing acceleration structure without modification. Reference [35] proposes a frame-by-frame intermittent tracking method that is driven by an actuator, which is used for the motion-free blurred video shooting of fast-moving objects. By controlling the frame and shutter timing of the camera to reduce the motion blur and by synchronizing the vibration with the free-vibration-type actuator, the motion blur can be reduced in free-view high-frame-rate video shooting.

#### 3.2.3. Weak-Contrast Targets

For the recognition and tracking of weakly contrasted targets, most algorithms require prior information about targets; otherwise, they would be affected by heavy noise clutter [36]. Reference [37] proposed a new method based on image fusion and mathematical morphology. Based on the description of the manipulatable pyramid, the original image is fused, and the target tracking of the fused image is realized via the mathematical morphology method. Reference [38] conducted an in-depth analysis of the background characteristics, weak target characteristics, and motion characteristics and proposed a moving average method. Based on foreground extraction, the difference calculation of adjacent frames that are related to the continuity of a moving target is conducted to eliminate the interference points and reduce the false alarm rate. The pretracking detection method proposed in Reference [39] operates directly on the original sensor signal without the need for a separate explicit detection stage. The probability density function of the target state is generated from the original pixel level, the probability indicator of the target presence is calculated, and the Bayesian particle filter is used to complete the target tracking. Reference [40] proposed a feedback neural network for weakly contrasted target motion tracking against a natural cluttered background. To form a feedback loop, the model delays the output time and forwards the feedback signal to the previous neural layer.

### 3.3. Occluded Targets and Fast-Moving Targets

In the course of UAV dynamic tracking, especially if fast movement occurs [41] and relabeling is necessary after the target is lost for a short time [42], the typical method determines the target area continuously through the video sequence [43]. Scholars at home and abroad have also proposed the correlation filter tracking algorithm [44] and the circular structure of tracking by detection with kernels (CSK) algorithm [45]. The tracking efficiency is high, but the tracking performance for multiscale targets is poor, and it is difficult to resume tracking of a missing target. To overcome this problem, reference [46] improved the scale-adaptive multifeature fusion (SAMF) algorithm on the basis of kernelized correlation filters (KCF) [47]. A multifeature (grayscale, histograms of oriented gradients (HOG), and color names (CN)) fusion method was used to realize feature complementation, and a multiscale search strategy was used to realize scale-adaptive tracking to increase the tracking accuracy. However, because the algorithm must conduct seven types of scale detection calculations, the speed is much lower than that of KCF. Reference [48] combines filter and context-aware information [49] and uses an intermittent learning method to enhance the network context awareness to increase the modeling performance of the network for occluded objects. In Reference [49], the frame with the best tracking results was used as the key frame in the follow-up tracking, which optimizes the quality of the training set and reduces the computational cost, thereby overcoming the poor robustness of the filter method in complex scenes.

Reference [50] used vector field guidance for multitarget tracking in aerial videos. By improving the vector field guidance method of a single UAV and defining a variable confrontation tracking track, the cooperative confrontation tracking of the UAV group on a moving target group is used to solve the problem of the visual range of the UAV when tracking multiple ground targets, which is suitable for processing aerial video images of a fast-moving target. To solve the problem of visual control of target tracking in visible light aerial photography, Reference [51] adopted a ground target tracking control strategy based on vision to realize the real-time tracking of aerial photography targets. Aiming at solving the regional cooperative search problem of multi-UAVs, Reference [52] described the changes in the environment and target state with the search process based on the search information graph model and established a motion model for the dynamic analysis of UAVs to ensure the accuracy of model prediction, thereby realizing the accurate tracking of complex targets with motion trajectories. To address the abnormal filter response caused by background interference in aerial video, a clipping matrix and regularization term were introduced in Reference [53] to expand the search area and suppress the distortion. The spatially regularized correlation filter (SRDCF) algorithm, which was proposed in Reference [54], adds spatial penalty terms on the basis of discriminative correlation filters (DCF) to solve for the boundary utility and realize superior performance in large-scale movement and complex scenes. However, the need to review used multiframe information in the tracking process creates a computational cost problem. The spatial-temporal regularized correlation filters (STRCF), proposed in Reference [55], add spatial and temporal regular terms on the basis of the problems encountered with SRDCF, and tracking requires only the information of the previous frame to ensure time efficiency. Most available filter algorithms attempt to introduce a predefined regularization to improve the learning relationship of the target object, but they are difficult to adapt to special scenarios in practice. To overcome this problem, Reference [56] proposed an online adaptive learning spatiotemporal regularization method. By introducing spatial local change information into spatial regularization, DCF can focus on the trusted part of the target object. The algorithm realizes satisfactory tracking performance on four aviation datasets. Reference [57] evaluated the target state by establishing an unscented Kalman filter based on a multi-interaction model, which reduces the network’s evaluation error of the moving target but also increases the computational consumption.

## 4. Target Tracking Algorithm Based on a Deep Learning Network

With the development of computer vision, many visual target tracking frameworks have been proposed and applied to aerial video target tracking. This section briefly introduces a tracking algorithm based on depth Features, a tracking algorithm based on a Siamese network and a target tracking algorithm based on an attention mechanism.

### 4.1. Depth Features

A deep learning network that is represented by a convolutional neural network (CNN) can automatically learn all the effective features of the target from many training sets, which not only effectively overcomes the background noise but also realizes satisfactory tracking performance [58,59].

**Figure 2:**
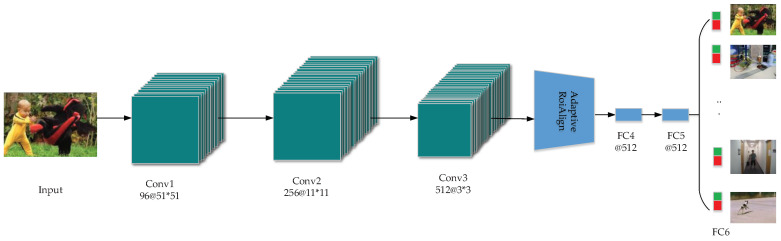
RT-MDNet structure. The model consists of K branches with a shared layer and a domain-specific layer. Green and red represent positive samples and negative samples, respectively, in each domain.

Reference [60] designed a lightweight CNN for learning the common attributes of a multidomain video to address scenarios such as target occlusion and target deformation in practical tracking. The network tracking structure uses online fine-tuning to improve the real-time performance of the tracking algorithm. Reference [61] added RoIAlign on this basis to accelerate feature extraction and classify internal targets through multitask loss, adding discriminative parameters to targets with similar semantics. The network structure is illustrated in Figure 2. First, the first three layers of convolution share the multiple-domain features learned by the network (e.g., the illumination change, motion blur, or robustness to size changes), and the adaptive RoIAlign extracts CNN features of each region of interest (RoI) to improve the feature quality and reduce the computational complexity. Layers FC4 and FC5 are mainly used to distinguish the background and the target, and the unique characteristics of each video domain are stored into the FC6 branch with softmax cross-entropy loss.

The online tracking process of the RT-MDNet algorithm is described in Algorithm 1.
**Algorithm 1** Online tracking process of RT-MDNet algorithm**Input:** Pretrained RT-MDNet convolution weights $w\{w_{i}\}$, where $w_{i}$ is the weight value of a convolution layer, and the initial target state $X^{l}$.**Output:** Adjusted target status $X^{*}$. 1.81:Random initialization of the last domain-specific layer weights $w_{6}$.2:Use bounding box regression technique to train boundary box regression function bbox.3:**for**: **do**4:    If (image == 1)5:    Acquire a convolution feature of the first frame image α(W).6:    else if7:    Acquire convolution features of the second frame and subsequent images $\alpha (w_{\gamma })$.8:    Draw a positive sample $S_{i}^{+}$ and a negative sample $S_{i}^{-}$.9:    Use $S_{i}^{+}$ and $S_{i}^{-}$ to update $w\left\{w_{j}\right\}:w=conv\left(S_{i}^{+},S_{i}^{-}\right),j=\left\{4,5,6\right\}$.10:    Set long-term update frame index $T_{l}^{i}$ and short-term update frame index $T_{S}^{i}$.11:    Draw target candidate sample state $X^{i}$.12:    Find the optimal state of the target position: $x^{*}=argmaxf^{+}\left(x^{i}\right)$, where $f^{+}\left(x^{i}\right)$ is the score of the target of the network evaluation.13:    if $f^{+}\left(x_{i}^{*}\right)\geq 0.5$, then draw a positive sample $S_{i}^{+}$ and a negative sample $S_{i}^{-}$,Long-term update frame index set $T_{l}=\sum_{i=1}^{n}T_{l}^{i}$, and short-term update frame index set $T_{S}=\sum_{i=1}^{n}T_{S}^{i}$.14:    if $T_{l}>t_{l}$, then $T_{l=}T_{l}/\left\{\min v\in t_{l}^{v}\right\}t_{l}$, where $t_{l}^{v}$ is the rate of change of the appearance of the long-term target.15:    if $T_{s}>t_{s}$, then $T_{s}=T_{s}/\left\{\min v\in t_{s}^{v}\right\}t_{s}$, where $t_{s}^{v}$ is the rate of change of the appearance of the short-term target.16:    Use bbox to adjust the optimal state of the target position: $x_{i}^{*}=bbox\left(x^{*}\right)$.17:    If (i%10 == 0)18:    then use $S_{V\in t_{l}}^{+}$ and $S_{V\in t_{s}}^{-}$ to update $w\left\{w_{j}\right\}:w=conv\left(S_{V\in t_{l}}^{+},S_{V\in t_{s}}^{+}\right)$.19:    else if $f^{+}\left(x_{i}^{*}\right)<0.5$20:    then use $S_{V\in t_{s}}^{+}$ and $S_{V\in t_{s}}^{-}$ to update $w\left\{w_{j}\right\}:w=conv\left(S_{V\in t_{s}}^{+},S_{V\in t_{s}}^{+}\right)$.21:**end for**

**Figure 3:**
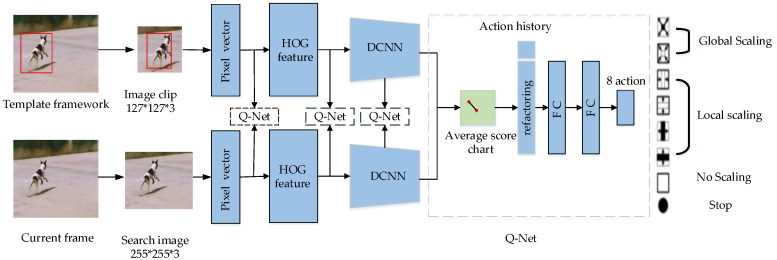
EArly Stopping Tracker (EAST) network structure. Judgment of the optimal feature layer by action.

Reference [62] proposed the EArly Stopping Tracker (EAST) to convert the adaptive tracking problem into a decision-making process. The network structure is illustrated in Figure 3. The network uses the offline reinforcement learning method to learn an agent for a single-frame image. Based on this agent, it decides to select a layer in a series of feature layers to realize target monitoring or to use the next layer to conduct the same processing. However, this method exhibits reduced accuracy with increasing speed.

The action selection process for the EAST network is described in Algorithm 2, where action_4 denotes four groups of actions, and action is an action(i) value.
**Algorithm 2** Action selection process for the EAST network**Input:** Feature map, action index: eigth_actionindex {}, the action value h${}_{l}$ from the first four layers, action list: $action\left\{action\left(i\right)\right\}(i\in 1,2...8)$.**Output:** Current conv layer action value. 1.81:Calculate the corresponding average value ${F_{l}}^{\prime }$ of the first N layers: ${F_{l}}^{\prime }=\sum_{k=1}^{l}F_{k}/l$.2:Construct the current state of the feature map:(${F_{l}}^{\prime },h_{l}$).3:Use vector merging to calculate the following feature sequence: $feature\_list={F_{l}}^{\prime }+action\_4$.4:Conduct feature reorganization of feature_list: *feature_map=fc(feature_list)*.5:Compare feature_map and eigth_actionindex, choose the action with the highest score: *sam_action=sam(feature_map,eigth_actionindex)*.6:if sam_action=Stop then “EAST”(early stop) at the subsequent target location will not be conducted.7:else then output the value of sam_action.

The discriminative correlation filter [63] shows substantial advantages in visual target tracking. The combination of a filter tracking framework and a deep neural network effectively improves the performance of the tracking algorithm [64,65]. Reference [66] proposed the multiple experts using entropy minimization (MEEM) algorithm within a tracking-by-detection framework to overcome the model drift caused by tracking failure or misalignment of the training samples. Aiming at solving this problem, the efficient convolution operators for tracking (ECO) algorithm was proposed in Reference [67], and continuous convolution operators (C-COT) [68] were simplified by modifying the number of model update frames, thereby reducing the model size, increasing the speed and reducing the risk of model overfitting. Simultaneously, according to the tracking results of the training set, components are generated by using the Gaussian mixture model (GMM) to ensure the diversity of the training set. However, the deep features of the network are not sufficiently effective, and the large amount of data calculation reduces the tracking speed of the network. Based on ECO, Reference [69] divided and conquered its depth features and shallow features, which substantially increased the robustness and tracking accuracy of the network structure.

To increase the network robustness, the multicue correlation filter tracking algorithm (MCCT), proposed in Reference [70], analyzes the fusion results that are obtained from the decision layers of multiple trackers to ensure the reliability of the results. The superimposed selection of adaptive strategies successfully distinguishes unreliable samples (in which there are occlusions or deformed data) to further avoid the problem of insufficient training due to sample contamination. Reference [56] combined the output of the Conv3 layer of the VGG-M [71] network with HOG-CN to increase the robustness of the model.

To overcome the difficulty of matching the training depth feature with the actual target information, the target-aware deep tracking (TADT) method, proposed in Reference [72], uses the global average of the backpropagation gradient to complete feature screening, evaluates the importance of each filter through a regression function, and applies a weighted supplement to the deep feature.

### 4.2. Siamese Network

To overcome the high computational burden and low speed of the previous deep neural network method, a Siamese network for introducing similarity learning into the matching process of the target image and search image was proposed, which balanced the costs of the tracking speed and tracking accuracy and gradually has become the preferred solution to the tracking problem [73,74].

Simplification of the target tracking problem to learn a common similarity mapping problem is an effective solution. The Siamese instance search for tracking (SINT) algorithm, proposed in Reference [75], learns a matching function through a Siamese network. The target feature of the first frame is used as a template, the subsequent sampling feature is matched with it for calculation, and the target with the highest score is selected as the final target. The algorithm uses a region pooling layer to realize model acceleration and demonstrates the feasibility of combining a deep neural network with traditional methods. Reference [76] also calculated the similarity between each position of the template and the image to be tested through template matching and selected the target with the highest similarity as the final target. The discriminative subspace learning model (DSLM) network, proposed in Reference [77], solves the problems of target occlusion and background interference by learning the relationship between the target module and the characteristics of the search area. Reference [78] constructed an asymmetric Siamese network (CFNet) that not only ensures the tracking accuracy but also simplifies the network structure. In Reference [79], DCF was used to complete the filtering, a probability heat map of the calculated result that was mapped to the target position was used to complete the online learning and tracking, and end-to-end training was realized.

These trackers simplify the problem of target tracking to the problem of learning a generic similarity map by learning the correlation between the feature representation of the target module and the search area. They do not consider the complex and changeable target scale, appearance or pixels in the actual tracking process. In Reference [80], tracking was decomposed into two parallel and collaborative threads—fast discriminative scale space tracking (FDSST) was used for fast tracking, and a Siamese network was used for accurate verification, thereby realizing both high accuracy and high speed. The Siamese region proposal network (SiamRPN) algorithm, which is proposed in Reference [81], overcomes the limitation of spatial invariance of the Siamese network. It is composed of a Siamese subnetwork and a region proposal subnetwork. The network completes the offline end-to-end training via large-scale image analysis, constructs a one-shot detection task to avoid time-consuming multiscale tests and obtains accurate candidate regions. SiamRPN increases the model accuracy and reduces the model size. DaSiamRPN, proposed in Reference [82], enriches the types of training data in the dataset via data augmentation, reduces the impacts of difficult negative samples on the network training, and improves the network generalization and discrimination performances. The interference recognition module in the network overcomes the low recognition accuracy caused by the lack of a self-updating model.

**Figure 4:**
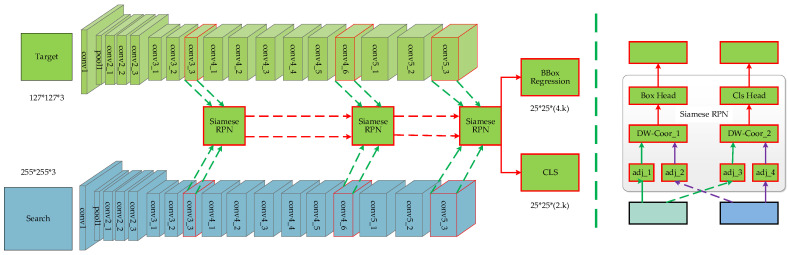
SiamRPN++ network structure. In the case of a specified target template and search area, the output intensive prediction is obtained by fusing the outputs of SiamRPN blocks. The middle siamrpn block is displayed on the right, which is divided into two parts: a classification branch and a boundary box regression branch.

The Siamese network is not a deep network due to the lack of translation-invariance. The SiamRPN++ algorithm, proposed in Reference [83] based on Reference [81], effectively solves this problem by modifying the sampling strategy. The network structure is illustrated in Figure 4. The method recombines the positioning features and deep semantic features obtained by ResNet and improves the feature expression performance according to the sequence of features from low to high, from small to large, and from thin to thick. The traditional image feature pyramid network (FPN) [84] is similar to it. For the loss of clipping invariance caused by padding, the model shifts the training sample labels to alleviate the centralization problem caused by the deep network.

The SiamRPN block of the SiamRPN++ algorithm is described in Algorithm 3.
**Algorithm 3** SiamRPN block**Input:** Feature map (φ(z),φ(x)), where φ(z) is the feature vector of the template frame; φ(x) is the feature vector of the detection frame.
**Output:** Classification results and regression results of bbox. 1.8
1:Use x as a convolution kernel on φ(z) to conduct the convolution operation to obtain the following anchor sequence: $A_{W*h*2k}^{cls}={\left[adj\_1\right]}_{cls}★{\left[adj\_2\right]}_{cls}$.2:Use z as a convolution kernel on φ(z) to conduct the convolution operation to obtain the following anchor sequence: $A_{W*h*4k}^{reg}={\left[adj\_3\right]}_{reg}★{\left[adj\_4\right]}_{reg}$.3:Calculate the positive sample sequence S${}^{+}$ and the negative sample sequence S${}^{-}$ by intersection over union (IoU) processing of all anchor sequences and the target real frame.4:Calculate the regression offset dx, dy, dw, dh of $A_{W*h*4k}^{reg}$ and binary classification {0,1} label of $A_{W*h*2k}^{cls}$.5:Reshape $A_{w*h*4k}^{reg}$.6:Conduct bbox regression using the smooth L1 loss: ${\mathrm{smooth}}_{L1}\left(x,\sigma \right)=\left\{\begin{array}{c} 0.5\sigma^{2}x^{2},|x|\leq \frac{1}{\sigma^{2}}\\ \left|x\right|-\frac{1}{2\sigma^{2}},\left|x\right|\geq \frac{1}{\sigma^{2}} \end{array}\right.$.7:Remove the anchor sequences with label=-1 from $A_{W*h*2k}^{cls}$.8:The cross-entropy function is used to calculate the classification results of the step 7 results.9:Output the regression results of bbox for step 6 and classification results for step 8.

Siam R-CNN, proposed in Reference [85], is a redetection architecture based on the trajectory dynamic programming algorithm (TDPA). Based on the Siamese framework, the self-motion and mutual motion of all potential objects are modeled, and the detected information is summarized into tracklets to complete the detection. This method is suitable for long-term tracking and is sufficient for addressing tracking failure after the target has been blocked for a long time. The Siamese box adaptive network (SiamBAN), proposed in Reference [86], simplifies the tracking problem into a problem of parallel classification and regression and directly conducts classification and regression operations on targets in a unified fully convolutional network (FCN). This avoids the computational complexity of the Siamese network due to the introduction of RPN and increases the network flexibility and generalization performance. The unsupervised deep tracker (UDT), proposed in reference [87], applies unsupervised learning to target tracking, uses three consecutive frames to evaluate the prediction deviation to increase the accuracy of the tracker, and applies a sensitive loss function to allocate a weight to each sample to overcome the noise caused by the random initialization of the target box in the unsupervised training.

### 4.3. Attention Mechanism

Challenges remain in ensuring the real-time performance and application of the tracker, and the available partial tracking algorithms cannot distinguish between the target and the background, which renders it difficult to address the changes of the target shape and background in real time. The attention mechanism module within the deep learning network reinforces important features in the image, thereby helping address issues such as target tracking failures [88].

**Figure 5:**
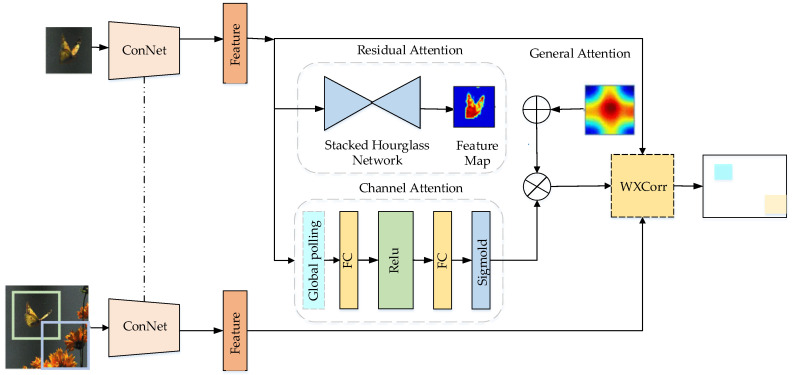
RASNet structure. The RASNet consists of a shared feature extractor, attention mechanisms (general attention, residual attention, and channel attention), and a weighted cross-correlation layer (WXCorr).

Reference [89] proposed the residual attentional Siamese network (RASNet) algorithm and reconstructed the filtering mode of the Siamese network based on a CNN, thereby effectively avoiding the overfitting problem. The algorithm separates representational learning from discriminant learning and enhances the discrimination performance and adaptability of the algorithm. Real-time tracking is realized. The network structure is illustrated in Figure 5, which contains three attention mechanisms. General Attention refers to the introduction of the attention mechanism to integrate the common features of targets and highlight the commonness of features. Residual attention considers differences in learning objectives. Channel attention adapts to various objectives and eliminates noise.

The attention fusion process of the RASNet algorithm is described in Algorithm 4.
**Algorithm 4** Attention Fusion Process of the RASNet algorithm**Input:** Feature map.**Output:** Trace box *q* with the largest response value. 1.81:The feature map is downsampled and upsampled by the residual attention mechanism to obtain the target semantic feature sequence: feature_R.2:The general attention mechanism is used to extract the information of multiframe feature maps, and the common feature sequence of the feature maps is obtained: feature_G.3:The dual attention feature is calculated: *feature_D = feature_R+feature_G*.4:Calculate channel weights: *channel_score=Sigmoid(Channel Attention(feature map))*.5:The fusion feature sequence is calculated: feature_list=feature_D⊗channel_score.6:The trace box *q* with the largest response value in the *feature_list* is identified via the weighted cross-correlation method: where α represents dual attention, β represents channel attention, *Z* represents a template image $f_{p^{\prime },q^{\prime }}=\sum_{i=0}^{m-1}\sum_{j=0}^{n-1}\sum_{c=0}^{d-1}\alpha_{i,j}\beta_{c\varphi i,j}$,$c\left(Z\right)\varphi_{p^{\prime }+i,q^{\prime }+j,c}\left(X\right)+b$ and *p* is a real box in *Z*, *X* represents the search image and *q* is the trace box in *X*.

**Figure 6:**
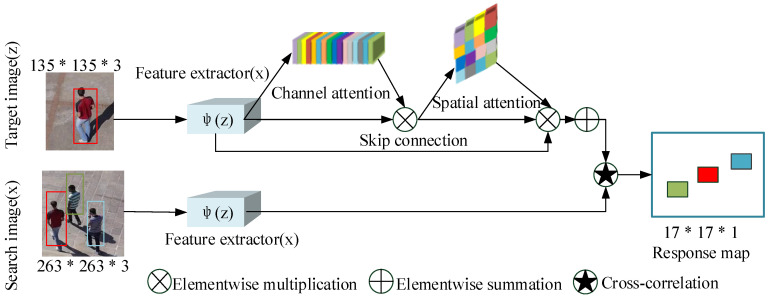
Spatial attention (SCSAtt) structure. The channel attention and spatial attention are combined to learn on “what” and “where” to concentrate or suppress target information, thereby effectively locating target information.

Reference [90] proposed spatial attention (SCSAtt), which ensured the model’s speed and increases its robustness. SCSAtt uses weight allocation to highlight the importance of the feature of the channel—namely, the channel attention module—and uses the spatial attention module to highlight the area with the most information on the feature diagram to determine the target location. The network structure is summarized in Figure 6.

The Channel-Spatial attention calculation process in the SCSAtt algorithm is described in Algorithm 5.
**Algorithm 5** Channel-Spatial attention calculation process in the SCSAtt algorithm**Input:** Feature map $F_{M}^{H*W*C}$.**Output:** Channel-Spatial attention Λ(ϕ(z)). 1.81:Use global max-pooling to obtain the $F_{M}$ object feature: Fmax1∗1∗C=fc2(ReLU(fc1(GPoolmax(FMH∗W∗C)))).2:Use global average-pooling to obtain the $F_{M}$:Favg1∗1∗C=fc2(ReLU(fc1(GPoolavg(FMH∗W∗C)))).3:Use elementwise summation to fuse two feature vectors:$\varphi_{c}{\left(\cdot \right)}^{1*1*C}=\sigma \left(F_{\max}^{1*1*C}⊕F_{\mathrm{avg}}^{1*1*C}\right)$.4:Calculate channel attention feature map $C_{A}$: $C_{A}=\varphi_{C}\left(F_{M}\right)⊗F_{M}$.5:Calculate $S_{\max}^{H*W*1}$ for $C_{A}$ with global max-pooling: $S_{\max}^{H*W*1}=GPool_{max}\left(C_{A}^{H*W*C}\right)$.6:Calculate $S_{\mathrm{avg}}^{H*W*1}$ for $C_{A}$ with global average-pooling: $S_{\mathrm{avg}}^{H*W*1}=GPool_{avg}\left(C_{A}^{H*W*C}\right)$.7:Calculate the spatial attention, $\vartheta^{3*3}$ is 3*3 convolution layer: $\varphi_{S}{\left(\cdot \right)}^{H*W*1}=\sigma (\vartheta_{1}^{3*3}\left(\mathrm{concat}\left[S_{\max}^{H*W*1},S_{\mathrm{avg}}^{H*W*1}\right]\right)$.8:Use the channel attention feature map to determine the ultimate effect on the spatial attention feature map S${}_{A}$:$S_{A}^{H*W*C}=\varphi_{S}{\left(\cdot \right)}^{H*W*1}⊗C_{A}^{H*W*C}$.9:Calculate the final stacked channel-spatial attention: $\Lambda \left(\phi z\right))=C_{A}⊕S_{A}$.

Similar to SCSAtt, the feature integrated correlation filter network (FICFNet) algorithm, proposed in reference [91], is a two-branch parallel connection network structure that unifies the three processes of feature extraction, feature integration and DCF learning. The feature integration module of the network cascades the shallow feature and the deep feature and uses the channel attention mechanism to adaptively combine the channel weight into the integrated feature, and the obtained target timing information can solve the problems of target occlusion and target deformation.

## 5. Experiment

### 5.1. Datasets

#### 5.1.1. Baseline Assessment

To accurately evaluate the model performance, experiments were conducted on aerial datasets UAV123 [11] and UAV20L [11]. UAV123 contains 123 fully annotated HD video sequences over 110K frames from the perspective of low-altitude aviation. Each video sequence has 12 attribute categories: Aspect Ratio Change(ARC), Background Clutter(BC), Camera Motion(CM), Fast Motion(FM), Full Occlusion(FOC), Illumination Variation(IV), Low Resolution(LR), Out-of-View(OV), Partial Occlusion(POC), Similar Object(SOB), Scale Variation(SC), and Viewpoint Change(VC). A video sequence may have a variety of attributes that are affected by the shooting conditions, and the frequency differs among the attributes. UAV20L is a subset of UAV123 and contains 20 long-video sequences. The UAV dataset has been tagged with the size and location information of the target in each video sequence and can be used for model initialization and model evaluation.

#### 5.1.2. Evaluating Indicators

**Figure 7:**
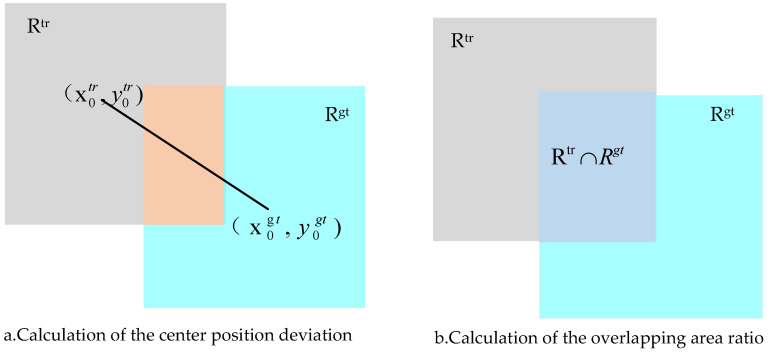
Evaluating indicators.

In this paper, two evaluation indicators, accuracy and success, are used to complete the quantitative analysis of the model. Accuracy refers to the percentage of the target center position error that is in the specified range, and the center position error is defined as the average Euclidean distance between the center position of the real box(x${}_{0}^{gt}$,y${}_{0}^{gt}$) and the center position of the tracking prediction box(x${}_{0}^{tr}$,y${}_{0}^{tr}$), as illustrated in Figure 7a. The proportion of the overlap scores (which is calculated from the intersection ratio) of the real box and the prediction box that exceed the threshold frames in the video timing sequence is the success degree, as presented in Figure 7b. The error of the center position is a widely used standard, which cannot be easily used to evaluate the performance of the tracker in the case of target loss. The accuracy curve is generated accordingly, and the corresponding value of 20 pixel points is adopted as the accuracy evaluation index [16]. When the center position error cannot be used to evaluate the target scale change, the performance of the tracker can be compensated by an evaluation index that is based on the area overlap ratio and is generated accordingly, as expressed in Formula (7).

(7) $$S=\frac{|R^{\mathrm{tr}}\cap R^{gt}|}{|R^{\mathrm{tr}}\cup R^{gt}|},$$

where *R*${}^{\mathrm{tr}}$ represents the real target boundary box, *R*${}^{gt}$ represents the prediction box of the tracking results, and ∪ and ∩ represent the union and intersection, respectively, of the two areas. This article uses the one-pass evaluation (OPE) accuracy and success graph to complete the model evaluation by ranking the tracking algorithms using the area under the curve (AUC) from the success graph. The parameter standards follow the default UAV123 settings.

The algorithm codes are implemented in the server with an NVIDIA TITAN V GPU by MATLAB and PYTHON, and the configuration parameters of the experimental environment are shown in Table 2.The codes of the trackers we reproduced are obtained from the GitHub repository, and the URLs are shown in Table 3. The training models of all tracking algorithms adopt the original models without retraining.

### 5.2. Evaluaton in UAV123

#### 5.2.1. Overall Evaluation

**Figure 8:**
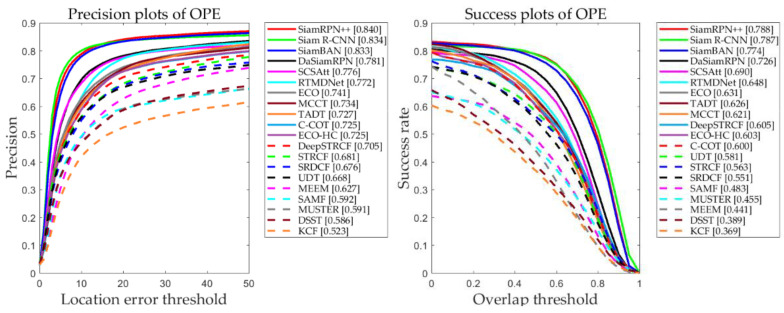
Overall accuracy and success rate of the trackers in the UAV123 benchmark test. The abscissa is the threshold, and the ordinate is the precision value.

In this paper, a total of 20 tracking algorithms are compared. Figure 8 presents the results for the algorithms on UAV123, which is the aerial photography dataset. Table 4 shows the characteristics of the tracking algorithm. Among them, SiamRPN++, SiamR-NN, SianBAN, SCSAtt, DaSiamRPN, and UDT are trackers that are based on Siamese networks. RT-MDNet, ECO, C-COT, MCCT, TADT, and DeepSTRCF are depth-based trackers. STRCF, SRDCF, MEEM, MUSTER, DSST, ECO-HC, KCF and SAMF are trackers that are based on handcrafted features. The trackers that are based on Siamese networks realize the best performances on the two measurement standards, with accuracy and success rates of 0.840 and 0.788, respectively, thereby outperforming the other tracking algorithms. This is a major breakthrough in tracking in the field of deep learning.

In the UVA 123 dataset, according to a comparison of the Siamese network model structures, SiamRPN++ utilizes a deep network, namely, ResNet, to fully extract target features by recombining features of shallow and deep layers. The network structure is relatively complex, but the advantage lies in the combination of a Siamese network and a deep structure to complete feature extraction. Siam R-CNN uses a Siamese network to apply the Faster R-CNN to solve the tracking problem and uses dynamic programming to address occlusion and target disappearance, which is suitable for long-term tracking and severely occluded scenes. However, the network structure is the most complex, and the computational burden is large. SiamBAN uses the representational capability of a fully convolutional network to simplify the tracking problem into classification and regression, thereby avoiding the hyperparameter problem. The accuracy and success rates of the SCSAtt tracker are 0.776 and 0.69, respectively; hence, the attention mechanism is an effective mechanism that helps the network increase the tracking accuracy. Since the structure of the DaSiamRPN algorithm cannot utilize deep features, there are gaps in the accuracy and success rates compared with the methods based on deep features, which demonstrates the importance of deep features. The UDT algorithm is the first unsupervised tracking algorithm to be implemented in a Siamese network framework, and its accuracy is consistent with that of SRDCF.

The trackers that are based on deep characteristics are being gradually optimized. While the tracking speed of RT-MDNet far exceeds that of ECO, it realizes the same success rate and accuracy as ECO; hence, the multidomain combination method is effective. By introducing deep features on the basis of the STRCF algorithm, the result of the DeepSTRCF algorithm is improved substantially compared with that of the STRCF algorithm.

Which models perform best?

Compared with other tracking algorithms, SiamRPN++, SiamBAN, and SCSATT networks have the best tracking performance, which can not only meet various challenges but also meet the real-time requirements. This is because these algorithms do not update the network parameters during online tracking, thus avoiding the time consumption caused by a large amount of computation.

Which models are more robust?

The Siam R-CNN algorithm uses the TDPA mechanism to address the problem of tracking failure after serious occlusion and target loss in online tracking, thus improving the robustness of the model. The ECO algorithm uses GMM to ensure the diversity of training sets and reduce the risk of model overfitting. DeepSTRCF improves the robustness of the model by fusing CNN features, HOG and CN. The MCCT algorithm comprehensively considers the tracking results of multiple trackers to ensure the reliability of the tracking results, and filters unreliable samples through an adaptive strategy to improve the robustness of the model.

Which models are lightweight?

The Siam-BAN algorithm simplifies the tracking problem to parallel classification and regression and directly classifies targets in FCN, which reduces the computational complexity and ensures a simple network structure and strong flexibility. The RT-MDNet algorithm simplifies the tracking problem to target recognition and achieves a higher tracking effect by considering the interference of similar objects in the loss function. The TADT algorithm assumes that the tracking task needs only the information of specific channels related to the target, eliminates other redundant channels, reduces the feature information used in the tracking process, and speeds up the tracking speed.

Which models are suitable for long-term tracking?

The DaSiamRPN algorithm improves the generalization ability of the model by enhancing the diversity of training samples, and uses a local-to-global strategy to solve the problem of target loss during long-term tracking.

#### 5.2.2. Attribute Evaluation

**Figure 9:**
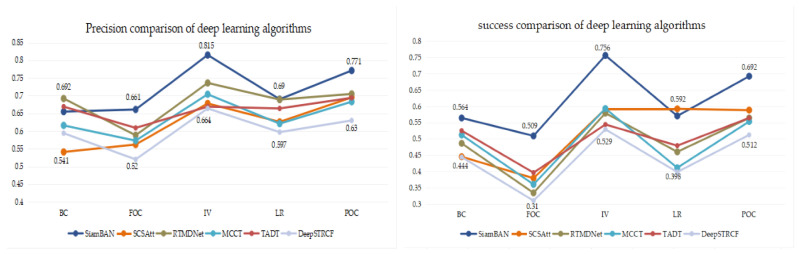
Result comparison of deep learning trackers.The abscissa is the attribute, and the ordinate is the precision value.

To fully evaluate the performance of the tracker in a variety of challenging scenarios, this article compares 12 different attributes in terms of accuracy and success on the UAV123 dataset. Table 5 and Table 6 presents the evaluation results of these attributes by all target tracking algorithms, and Figure 9 compares the methods that are based on deep learning. According to the experimental results, the trackers based on Siamese networks can effectively handle various challenging scenes; for the scenes in the categories of Aspect Ratio Change (ARC), Camera Motion (CM), Illumination Variation (IV) and Viewpoint Change (VC), the results are especially outstanding. Hence, the Siamese network structure performs satisfactorily in solving tracking problems such as target scale change, target rapid motion and target background similarity interference. In addition, compared with the “attention mechanism” approach, the “deep feature tracker” approach performs better in the categories of Background Clutter (BC), Full Occlusion (FOC), Low Resolution (LR) and Partial Occlusion (POC), thus, rich depth features can well overcome the problems of target occlusion and deformation.

**Figure 10:**
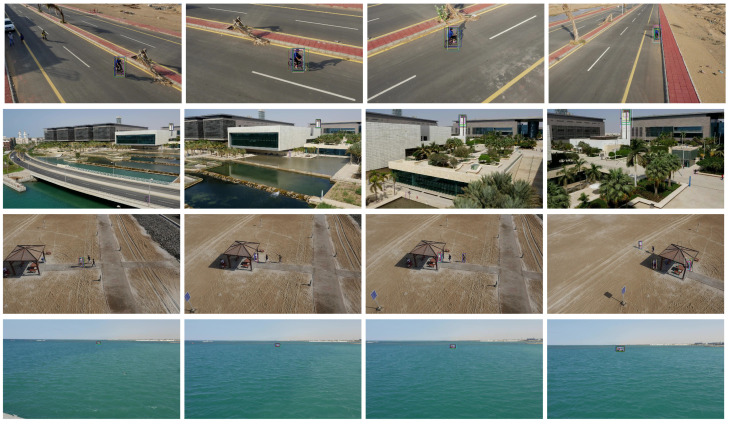
Visualization of tracking results in different test sequences. The test sequence included bicycles, boats, buildings and people.

The visualization results of each tracker on the aerial photography dataset UAV123 are shown in Figure 10. Among them, the first line is the tracking result of the video sequence bike, the second line is the tracking result of the video sequence building, the third line is the tracking result of the video sequence group, and the fourth line is the tracking result of the video sequence boat. We can see in Figure 10 that under the condition of a simple background, as in bike, the trackers show good tracking effects. However, when the background contains objects similar to the target, as in building and group, the background is seriously affected by interference, and some trackers encounter difficulty distinguishing the target from similar objects. We can also see that when the target is severely occluded or temporarily disappeared, as in group, the trackers fail to track. When the target size is small, as in boat, due to the small proportion of the target in the image, it is difficult to obtain features, and the tracking accuracy of some trackers is poor.

**Figure 11:**
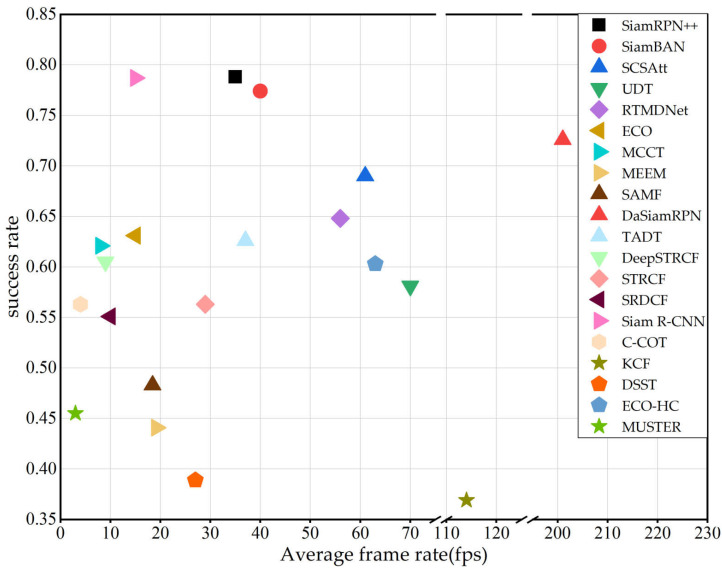
The success rate and frame rate of trackers on the UAV123 dataset.

The speed comparison among all the trackers is shown in Figure 11 where the success rate vs. fps is plotted for the UAV123 dataset.Compared with other algorithms, SN-based trackers have higher frame rate, This is because the network parameters are not updated during online tracking. Among the CNN-based trackers, RTMDNet has the highest frame rate and outperforms the other CNN-based trackers. This is because RTMDNet adds an adaptive ROI layer between the convolution layer and the full connection layer. This method can greatly reduce the computational complexity of the tracking process and enable it to achieve higher frame rate in the tracking process. Among the CF-based trackers, ECO has the highest frame rate and outperforms. The factorized convolution operation makes the tracker more efficient, enabling it to achieve higher frame rate and better performance. ECO-HC using only hand-crafted features (HOG and Color Names), thus further reducing the computation of the model, thereby allowing it to achieve a higher fps than ECO. It is also seen that the KCF has a high fps, but it has the lowest success rate due to the tracker only extracts HOG features.

### 5.3. Evaluation in UAV20L

**Figure 12:**
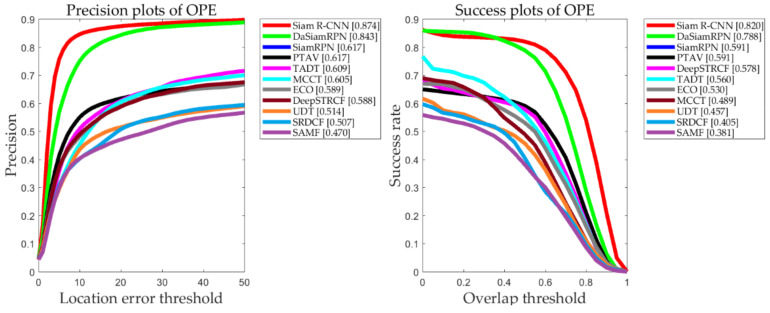
Overall accuracy and success rates of the trackers in the UAV20L benchmark test.

UAV20L is a representative aerial long-video dataset. This paper compares the performances of 10 representative long-video trackers. According to the evaluation report in Figure 12, Siamese network trackers still perform at a high level and far surpass other trackers that are based on depth characteristics. In addition, we analyzed the evaluation results of 12 independent attributes that were provided by UAV20L: Aspect Ratio Change (ARC), Background Clutter (BC), Camera Motion (CM), Fast Motion (FM), Full Occlusion (FOC), Illumination Variation (IV), Low Resolution (LR), Out-of-View (OV), Partial Occlusion (POC), Similar Object (SOB), Scale Variation (SC), and Viewpoint Change (VC). Table 7 and Table 8 present the evaluation results of these attributes by all target tracking algorithms and presents the scores of the 10 trackers on these attributes.

The Siamese network trackers perform better on Scale Variation (SV), Aspect Ratio Change (ARC), Fast Motion (FM), Partial Occlusion (POC), Out-of-View (OV), Viewpoint Change (VC), Camera Motion (CM), and Similar Object (SOB). For Background Clutter (BC), Full Occlusion (FOC), Illumination Variation (IV) and Low Resolution (LR). The deep neural network trackers show unique advantages. Among them, the MCCT algorithm uses an adaptive strategy to remove contaminated samples. It is effective in working with background interference and realizes a success rate nearly 20% higher than that of the Siamese network. The TADT algorithm uses a callback function to ensure that the deep convolutional network retains the positioning features of the target after convolution learning to cope with complete occlusion and low resolution. The Full Occlusion(FOC) success rate is 0.307, and the Low Resolution(LR) success rate is 0.432, which exceeds that of Siam R-CNN by 6%.

### 5.4. Comparison and Summary

For a single target, the available tracking algorithms are relatively mature when the motion trajectory and background are relatively simple, and better results can be obtained by using filters, deep learning and other methods. For the problem of multicamera collaborative tracking, methods of combining geographic information have been proposed, but they still cannot solve the problem of multi-man-machine collaborative tracking of multiple targets in complex scenarios. Table 9 summarizes and compares 35 aerial photography target tracking algorithms with better performance.

For aerial photography target tracking with various ranges, environments and targets, both the tracking speed and the recognition accuracy must be considered. Therefore, the methods discussed in this paper can be divided into two categories: those that realize increased accuracy and those that realize increased tracking speed. Target position information can be used to establish a motion model that has a fast tracking speed, but the accuracy of tracking is poor; when tracking is implemented by model matching, the tracking accuracy is high, but the processing speed is slower. Due to the successful application of the correlation filtering algorithm in the single target tracking field, the algorithm transforms the data processing from the real domain into the frequency domain, and the processing speed is substantially increased. Therefore, for a single target with a relatively simple motion trajectory and background, the available target tracking algorithms and technologies are relatively mature, and the method combining filtering and deep learning can yield superior results.

Compared with the traditional method of correlation filtering, target tracking based on deep learning realizes substantial improvements in terms of accuracy and detection speed, especially the network structure based on Siam. However, due to the strong dependence of deep learning on data and the insufficient amount of data in target tracking, the current framework cannot yield satisfactory results, and the explanatory performances of related methods of deep learning is insufficient. To summarize the available target tracking algorithms, we still must overcome the following challenges.

1. Changes in the target attitude. Multiple postures of the same moving target reduce the accuracy of target recognition, which is a common interference problem in target tracking. When the target attitude changes, its characteristics differ from those at the original attitude, and the target is easily lost, thereby resulting in tracking failure. An attention mechanism can help networks focus on important information regarding targets and reduce the probability of target loss during tracking. The utilization by deep learning network algorithms of an attention mechanism to ensure the accurate positioning of network targets is a promising research direction.
2. Long-term tracking. In a long-time tracking process, due to the height and speed limit of aerial photography, the tracking target scale in the images in the video change with increasing tracking time. Since the tracking box cannot utilize adaptive tracking, it contains redundant background feature information, thereby leading to parameter update error of the target model. In contrast, the accelerated flight causes the target scale to increase continuously. Since the tracking box cannot contain all characteristic information of the target, parameter update error also occurs. According to the experimental results of this paper, the Siamese network realizes satisfactory performance in long-term tracking but cannot conduct online real-time tracking. The construction of a suitable long-term target tracking model according to the characteristics of long-term tracking tasks and their connection points with short-term tracking that combines the depth characteristics and migration learning remains a substantial challenge.
3. Target tracking in a complex background environment. Against a complex background such as night, substantial changes in illumination intensity or too much occlusion, the target exhibits reflection, occlusion or transient disappearance during movement. If the moving target is similar to the background, tracking failure will occurs because the corresponding model of the target cannot be found. The main strategies for solving the occlusion problem are as follows: The depth characteristics of the target can be fully extracted to ensure that the network can handle the occlusion problem. During the offline training, occluded targets can be added into the training samples so that the network can fully learn coping strategies when a target is blocked and the trained offline network can be used to track the target. Multi-UAV collaborative tracking can utilize target information from multiple angles and effectively solve the problem of target tracking against a complex background.
4. Real-time tracking. Real-time tracking is always a difficult problem in the field of target tracking. The current tracking method based on deep learning has the advantage of learning from a large amount of data. However, in the target tracking process, only the annotation data of the first frame are completely accurate, and it is difficult to extract sufficient training data from the network. The network model of deep learning is complex and has many training parameters. If the network is adjusted online in the tracking stage to ensure the tracking performance, the network tracking speed is severely affected. Large-scale datasets obtained via aerial photography are gradually becoming available, which include rich target classes and involve various situations that are encountered in practical applications. Many tracking algorithms have continued to learn depth characteristics from these datasets via an end-to-end approach, which is expected to further enable target tracking algorithms to realize real-time tracking while ensuring satisfactory tracking speed.

## 6. Future Directions

### 6.1. Cooperative Tracking and Path Planning of Multiple Drones

As the sensing field of a single UAV is limited and the 3D feature information of the target and scene is lost, it is necessary to cooperatively utilize multiple UAVs. However, in multiple-UAV cooperative tracking, since the information surveillance camera is discrete, there is a lack of information for the rapid integration mechanism among multiple cameras, and multicamera coordination is necessary for efficient target tracking [92,93]. Thus, the problem of cooperative path planning [94] is also encountered. Although satisfactory planning and design results have been obtained, multiple challenges are faced, such as challenges regarding locally optimal solutions [95] and the iteration time [96].

### 6.2. Long-Term Tracking and Abnormal Discovery

With the frequent occurrence of abnormal events in public areas, technology for the detection of abnormal crowd behavior based on aerial video has become a research hotspot at home and abroad in recent years [97]. Long-time tracking and monitoring are required, which pose new challenges in aerial photography tracking. In terms of degree, abnormal events can be divided into two groups: abnormal group events and abnormal individual events [98]. These events must have occurred during the process of tracking the abnormal behavior detection alarm. The use of target behavior prediction and security situational awareness to realize real-time anomaly warning is the key problem to be solved in the future.

### 6.3. Visualization and Intelligent Analysis of Aerial Photography Data

UAVs rely on a variety of wireless network technologies to realize real-time video surveillance and air transfer of related images or videos to a mobile command platform or background system for intelligent identification and analysis and to provide a decision-making basis for manpower deployment, emergency response and technical support. However, due to the lack of corresponding technical support and solutions, information sharing among aerial video equipment to establish and improve the aerial video application integration platform is not convenient, which constrains the role of the intelligent monitoring system in public security. Based on intelligent analysis, with the deployment of the 5G network, the realization of real-time tracking and security situational awareness prediction via a visual approach is essential for the future application of the visualization platform.

## Figures and Tables

**Table 1:** Common aerial video datasets.

Datasets	Number of Videos	Shortest Video Frames	Average Video Frames	Longest Video Frames	Total Video Frames
UAV123 [11]	120	109	915	9085	112,578
UAV20L [11]	20	1717	2934	5527	58,670
ALOV300++ [12]	314	19	483	5975	151,657
VOT-2014 [13]	25	164	409	1210	10,000
VOT-2017 [14]	60	41	356	1500	21,000
OTB2013 [15]	51	71	578	3872	29,491
OTB2015 [16]	100	71	590	3872	59,040
Temple Color 128 [17]	129	71	429	3872	55,346
LaSOT [18]	1400	1000	2506	11,397	3.52 M
NFS [19]	100	169	3830	20,665	383,000
VisDone 2018 [20]	288	-	10,209	-	261,908

**Table 2:** Configuration parameters of experimental environment.

Parameter Name	Version or Value
Operating system	Windows 10
CPU	Intel Xeon 3.60 GHz
GPU	NVIDIA TITAN V/12 G
CUDA	CUDA10.1
RAM	32 GB

**Table 3:** The URLs of the implemented tracking algorithm code. P represents the implementation in python and M represents the implementation in matlab.

Tracker	Publication	Year	Code	Code Repository
SiamRPN++	CVPR	2019	P	https://github.com/PengBoXiangShang/SiamRPN_plus_plus_PyTorch
SiamBAN	CVPR	2020	P	https://github.com/hqucv/siamban
Siam R-CNN	CVPR	2020	P	https://github.com/VisualComputingInstitute/SiamR-CNN
DaSiamRPN	ECCV	2018	p	https://github.com/foolwood/DaSiamRPN
SCSAtt	IEEE	2020	P	https://github.com/maklachur/SCSAtt
UDT	CVPR	2019	P	https://github.com/594422814/UDT
RT-MDNet	ECCV	2018	P	https://github.com/HyeonseobNam/py-MDNet
ECO	CVPR	2017	M	https://github.com/martin-danelljan/ECO
ECO-HC	CVPR	2017	M	https://github.com/martin-danelljan/ECO
C-COT	ECCV	2016	M	https://github.com/martin-danelljan/Continuous-ConvOp
MCCT	CVPR	2018	M	https://github.com/594422814/MCCT
TADT	CVPR	2019	M	https://github.com/XinLi-zn/TADT
DeepSTRCF	CVPR	2018	M	https://github.com/lifeng9472/STRCF
MEEM	ECCV	2014	M	http://cs-people.bu.edu/jmzhang/MEEM/MEEM.html
STRCF	CVPR	2018	M	https://github.com/lifeng9472/STRCF
SRDCF	ICCV	2015	M	https://github.com/JHvisionchen/SRDCF-matlab
SAMF	ECCV	2015	M	https://github.com/ihpdep/samf
MUSTER	CVPR	2015	M	https://sites.google.com/site/zhibinhong4131/Projects/muster
DSST	BMVC	2014	M	http://www.cvl.isy.liu.se/en/research/objrec/visualtracking/scalvistrack/index.html
KCF	IEEE	2014	M	http://www.robots.ox.ac.uk/~joao/circulant/

**Table 4:** Tracker characteristics.

Tracker	Base Network	Feature	Online-Learning	Real-Time
SiamRPN++	SiamRPN	CNN	N	Y
SiamBAN	SiamFC	CNN	N	Y
Siam R-CNN	SiamFC	CNN	Y	N
DaSiamRPN	SiamRPN	CNN	Y	Y
SCSAtt	SiamFC	CNN	N	Y
UDT	SiamFC	CNN	N	Y
RTMDNet	MDNet	CNN	Y	Y
ECO	C-COT	CNN, HOG, CN	Y	N
ECO-HC	C-COT	HOG, CN	Y	N
C-COT	C-COT	CNN	N	N
MCCT	DCF	CNN	Y	N
TADT	TADT	CNN	N	Y
DeepSTRCF	STRCF	CNN, HOG, CN	Y	N
MEEM	MEEM	CNN	Y	N
STRCF	SRDCF	HOG, CN, Gray	Y	N
SRDCF	SRDCF	HOG, CN	Y	N
SAMF	KCF	HOG, CN, Gray	N	N
MUSTER	MUSTER	HOG, CN	N	N
DSST	CF	HOG, CN, Gray	N	N
KCF	CF	HOG	N	N

**Table 5:** The precision results of various trackers under the UAV123 dataset attribute. The best-performing tracker is displayed in red, and the second-best performer is in yellow.

Tracker	ARC	BC	CM	FM	FOC	IV	LR	OV	POC	SOB	SV	VC
Siam R-CNN	0.854	0.714	0.889	0.822	0.776	0.809	0.706	0.839	0.809	0.812	0.828	0.875
SiamBAN	0.796	0.645	0.848	0.805	0.671	0.766	0.719	0.789	0.765	0.777	0.813	0.824
SiamRPN++	0.818	0.655	0.863	0.774	0.661	0.815	0.690	0.816	0.771	0.800	0.820	0.876
DaSiamRPN	0.756	0.668	0.786	0.737	0.633	0.710	0.663	0.693	0.701	0.747	0.754	0.753
SCSAtt	0.722	0.541	0.775	0.690	0.562	0.678	0.626	0.721	0.695	0.78	0.749	0.747
ECO	0.654	0.624	0.721	0.652	0.576	0.710	0.683	0.590	0.669	0.747	0.707	0.680
RTMDNet	0.720	0.689	0.767	0.641	0.579	0.723	0.689	0.659	0.700	0.754	0.735	0.702
MCCT	0.683	0.616	0.720	0.614	0.573	0.704	0.621	0.659	0.683	0.741	0.700	0.681
TADT	0.667	0.669	0.723	0.617	0.609	0.669	0.664	0.626	0.694	0.728	0.692	0.655
DeepSTRCF	0.644	0.594	0.696	0.586	0.520	0.664	0.597	0.618	0.630	0.717	0.667	0.640
UDT	0.618	0.516	0.654	0.600	0.474	0.599	0.585	0.580	0.578	0.668	0.639	0.599
SRDCF	0.587	0.526	0.627	0.524	0.501	0.600	0.579	0.576	0.608	0.678	0.639	0.593
STRCF	0.586	0.563	0.658	0.5554	0.488	0.538	0.589	0.570	0.587	0.648	0.643	0.581
ECO-HC	0.653	0.608	0.712	0.587	0.569	0.653	0.631	0.599	0.653	0.698	0.690	0.640
C-COT	0.586	0.502	0.658	0.554	0.487	0.536	0.584	0.388	0.587	0.648	0.643	0.581
MEEM	0.563	0.516	0.595	0.418	0.460	0.509	0.580	0.476	0.526	0.629	0.591	0.680
SAMF	0.497	0.530	0.558	0.402	0.458	0.524	0.539	0.469	0.506	0.611	0.541	0.518
MUSTER	0.516	0.581	0.570	0.406	0.463	0.489	0.527	0.296	0.495	0.629	0.552	0.537
DSST	0.482	0.500	0.520	0.367	0.406	0.524	0.475	0.256	0.505	0.604	0.538	0.502
KCF	0.424	0.454	0.483	0.300	0.374	0.418	0.436	0.386	0.451	0.578	0.471	0.436

**Table 6:** The successful results of various trackers under the UAV123 dataset attribute. The best-performing tracker is displayed in red, and the second-best performer is in yellow.

Tracker	ARC	BC	CM	FM	FOC	IV	LR	OV	POC	SOB	SV	VC
SiamR-CNN	0.795	0.648	0.839	0.753	0.638	0.765	0.614	0.772	0.738	0.749	0.778	0.842
SiamRPN++	0.751	0.564	0.804	0.706	0.509	0.756	0.570	0.728	0.692	0.721	0.761	0.832
SiamBAN	0.724	0.549	0.783	0.723	0.510	0.699	0.590	0.707	0.678	0.695	0.746	0.772
DaSiamRPN	0.680	0.574	0.738	0.660	0.464	0.653	0.524	0.631	0.625	0.659	0.692	0.709
SCSAtt	0.597	0.445	0.691	0.564	0.379	0.592	0.592	0.600	0.588	0.673	0.655	0.645
ECO	0.497	0.479	0.599	0.463	0.358	0.534	0.470	0.506	0.548	0.629	0.588	0.530
RTMDNet	0.524	0.463	0.608	0.454	0.326	0.574	0.464	0.553	0.596	0.617	0.622	0.536
MCCT	0.521	0.512	0.618	0.464	0.360	0.593	0.411	0.543	0.553	0.615	0.578	0.546
TADT	0.501	0.525	0.613	0.456	0.396	0.544	0.479	0.499	0.564	0.610	0.582	0.513
DeepSTRCF	0.503	0.444	0.605	0.427	0.318	0.529	0.398	0.513	0.512	0.601	0.560	0.519
UDT	0.499	0.422	0.569	0.480	0.308	0.499	0.499	0.500	0.482	0.563	0.548	0.481
SRDCF	0.431	0.401	0.545	0.366	0.301	0.457	0.359	0.465	0.468	0.532	0.510	0.441
STRCF	0.418	0.425	0.512	0.359	0.289	0.385	0.388	0.470	0.469	0.550	0.516	0.426
ECO-HC	0.491	0.459	0.598	0.414	0.368	0.511	0.404	0.520	0.525	0.585	0.561	0.476
C-COT	0.584	0.382	0.539	0.357	0.289	0.381	0.382	0.471	0.462	0.547	0.510	0.421
MEEM	0.362	0.389	0.426	0.242	0.258	0.360	0.304	0.329	0.380	0.516	0.405	0.357
SAMF	0.362	0.408	0.450	0.283	0.249	0.362	0.269	0.349	0.392	0.500	0.430	0.354
MUSTER	0.516	0.439	0.432	0.243	0.242	0.354	0.296	0.297	0.347	0.471	0.405	0.385
DSST	0.482	0.389	0.346	0.200	0.226	0.331	0.256	0.293	0.342	0.401	0.322	0.299
KCF	0.422	0.341	0.347	0.187	0.210	0.296	0.210	0.257	0.321	0.379	0.307	0.283

**Table 7:** The precision results of various trackers under the UAV20L dataset attribute. The best-performing tracker is displayed in red, and the second-best performer in yellow.

Tracker	ARC	BC	CM	FM	FOC	IV	LR	OV	POC	SOB	SV	VC
Siam R-CNN	0.522	0.191	0.597	0.642	0.349	0.439	0.521	0.641	0.578	0.683	0.597	0.561
DaSiamRPN	0.517	0.191	0.595	0.641	0.346	0.436	0.520	0.637	0.572	0.667	0.584	0.558
SiamRPN	0.514	0.190	0.596	0.642	0.351	0.437	0.518	0.641	0.574	0.678	0.581	0.549
MCCT	0.516	0.382	0.54	0.534	0.418	0.563	0.475	0.575	0.573	0.618	0.586	0.495
ECO	0.489	0.382	0.567	0.493	0.409	0.551	0.486	0.546	0.554	0.559	0.567	0.507
TADT	0.521	0.383	0.588	0.614	0.444	0.518	0.550	0.534	0.577	0.587	0.588	0.505
PTAV	0.489	0.382	0.567	0.493	0.409	0.551	0.486	0.546	0.554	0.559	0.567	0.507
DeepSTRCF	0.488	0.381	0.566	0.508	0.429	0.523	0.512	0.549	0.556	0.563	0.566	0.503
UDT	0.446	0.378	0.496	0.492	0.427	0.437	0.445	0.478	0.487	0.521	0.489	0.402
SRDCF	0.389	0.252	0.482	0.327	0.331	0.411	0.429	0.495	0.491	0.522	0.481	0.414
SAMF	0.382	0.330	0.443	0.308	0.351	0.416	0.419	0.384	0.445	0.457	0.443	0.363

**Table 8:** The successful results of various trackers under the UAV20L dataset attribute. The best-performing tracker is displayed in red, and the second-best performer in yellow.

Tracker	ARC	BC	CM	FM	FOC	IV	LR	OV	POC	SOB	SV	VC
Siam R-CNN	0.490	0.137	0.569	0.544	0.241	0.431	0.432	0.623	0.549	0.691	0.691	0.57
DaSiamRPN	0.489	0.131	0.564	0.541	0.225	0.430	0.424	0.605	0.543	0.687	0.691	0.552
SiamRPN	0.483	0.136	0.557	0.537	0.238	0.427	0.416	0.618	0.533	0.682	0.678	0.561
MCCT	0.403	0.327	0.463	0.347	0.285	0.428	0.337	0.448	0.456	0.563	0.563	0.497
ECO	0.42	0.288	0.506	0.321	0.267	0.498	0.341	0.501	0.495	0.565	0.565	0.51
TADT	0.464	0.321	0.537	0.445	0.307	0.504	0.432	0.448	0.525	0.591	0.591	0.563
PTAV	0.42	0.288	0.506	0.321	0.267	0.498	0.341	0.501	0.495	0.565	0.565	0.51
DeepSTRCF	0.474	0.297	0.556	0.397	0.286	0.531	0.408	0.552	0.545	0.61	0.61	0.556
UDT	0.4	0.319	0.456	0.404	0.309	0.43	0.349	0.433	0.441	0.514	0.514	0.43
SRDCF	0.305	0.203	0.384	0.207	0.214	0.327	0.24	0.407	0.383	0.463	0.463	0.39
SAMF	0.281	0.268	0.349	0.143	0.22	0.37	0.275	0.307	0.356	0.371	0.371	0.349

**Table 9:** Comparison of aerial video tracking methods.

Category	Method	Applicable Target	Applicable Scenario	Number of Targets
Manual features	ASLA [22]	Common objectives	Severe target occlusion	Single target
	MUSTer [23]	Common objectives	Short/long-time tracking	Single target
	Characteristics of the cascade [62]	Common objectives	Hover aerial shot	Single target
	Moving average method [38]	Weak small targets	Smaller target	Single target
	Grayscale features, spatial features [35]	Weak/background similar targets	Complex background/small target	Single target
Filter tracking	Bayesian trackers [39]	Blurred objectives	Common scenario	Many objectives
	Wiener filtering [32]	Blurred objectives	Blurred target	Single target
	Vector field characteristics [50]	Fast/multitarget	Fast-moving speed/wide field of vision	Many objectives
	Feedback ESTMD [40]	Moving small target	Complicated background	Single target
	ARCF [53]	Moving target	Severe occlusion/background interference	Single target
	DSST [41]	Moving target	Common scenario	Single target
	KCF [47]	Moving target	Common scenario	Single target
	SRDCF [54]	Moving target	Large range of motion/complex scenes	Single target
	STRCF [55]	Moving target	Common scenario	Single target
	AutoTrack [56]	Moving target	Common scenario	Single target
Scale estimate	SAMF [46]	Moving target	Scale change	Single target
Depth features	RT-MDNet [61]	Moving target	Complicated background	Single target
	MEEM [66]	Multiscale target	General background	Single target
	C-COT [68]	Common objectives	General background	Single target
	ECO [67]	Common objectives	General background	Single target
	ECO+ [69]	Common objectives	Background complex/multiscale	Single target
	MCCT [70]	Common objectives	Target occlusion/complex background	Single target
	TADT [72]	Target deformation	Background interference/common scenario	Single target
	DeepSTRCF [55]	Similar objectives	Common scenario	Single target
Siamese network	SiamFC [76]	Target deformation	General background	Single target
	PTAV [80]	Common objectives	Common scenario	Single target
	SiamRPN [81]	Weak small targets	Common scenario	Single target
	Da SiamRPN [82]	Moving target	Long track	Single target
	SiamRPN++ [83]	Moving target	Various scenarios	Single target
	Siam R-CNN [85]	Multiscale target	Severe occlusion/common scenario	Single target
	SiamBAN [86]	Common objectives	Various scenarios	Single target
	UDT [87]	Multiscale target	Severe occlusion	Single target
Attention mechanism	RASNet [89]	Common objectives	General background	Single target
	SCSAtt [90]	Common objectives	Target scales vary substantially	Single target
	FICFNet [91]	Moving target	Severe deformation/occlusion of the target	Single target
